# Supplementary material for: Regulatory network structure determines patterns of intermolecular epistasis
Source: eLife. 2017 Nov 13;6:e28921. doi: 10.7554/eLife.28921 (PMC5699867; doi:10.7554/eLife.28921)
Supplement: Figure 2—source data 3. — P values of the differences in entropies of mutant libraries were calculated using a nonparametric permutation test. [file elife-28921-fig2-data3.pdf]

	low mutation probability *cis*	intermediate mutation probability *cis*	high mutation probability *cis*	low mutation probability *trans*	intermediate mutation probability *trans*	high mutation probability *trans*	low mutation probability system	intermediate mutation probability system	high mutation probability system
low mutation probability *cis*		$< 10^{-6}$	$< 10^{-6}$	$< 10^{-6}$	$< 10^{-6}$	$< 10^{-6}$	$< 10^{-6}$	$< 10^{-6}$	$< 10^{-6}$
intermediate mutation probability *cis*			$< 10^{-6}$	$< 10^{-6}$	$< 10^{-6}$	$< 10^{-6}$	$< 10^{-6}$	$< 10^{-6}$	$< 10^{-6}$
high mutation probability *cis*				$< 10^{-6}$	$< 10^{-6}$	$< 10^{-6}$	$< 10^{-6}$	$< 10^{-6}$	$< 10^{-6}$
low mutation probability *trans*					$< 10^{-6}$	$< 10^{-6}$	$< 10^{-6}$	$< 10^{-6}$	$< 10^{-6}$
intermediate mutation probability *trans*						$< 10^{-6}$	$< 10^{-6}$	0.00239	$< 10^{-6}$
high mutation probability *trans*							$< 10^{-6}$	$< 10^{-6}$	$< 10^{-6}$
low mutation probability system								$< 10^{-6}$	$< 10^{-6}$
intermediate mutation probability system									$< 10^{-6}$
high mutation probability system									
