# Supplementary material for: Regulatory network structure determines patterns of intermolecular epistasis
Source: eLife. 2017 Nov 13;6:e28921. doi: 10.7554/eLife.28921 (PMC5699867; doi:10.7554/eLife.28921)
Supplement: Figure 2—source data 4. — Comparison of random subsamples of each dataset to the full dataset using K-S test shows that observed distributions are not sensitive to reductions in sample size or mutant library diversity. We take 50 random subsamples for each subsample size of each cis- and trans-element mutant library in each relevant environment (both absence and presence of CI for cis-element libraries, only presence of CI for trans -element libraries). D-statistic from the K-S test is used to estimate significance (p value). [file elife-28921-fig2-data4.pdf]

Mutant library	Environment	Subsample size (x1,000)	D statistic	P value
Low mutation number *cis*	Absence of CI	50	0.0035	0.61
		100	0.0019	0.89
		250	0.0011	0.97
		500	$8x10^{-4}$	0.99
	Presence of CI	50	0.0026	0.90
		100	0.0024	0.70
		250	0.0012	0.94
		500	0.001	0.93
Intermediate mutation number *cis*	Absence of CI	50	0.0032	0.32
		100	0.0019	0.49
		250	0.0023	0.96
		500	$6x10^{-4}$	0.99
	Presence of CI	50	0.002	0.99
		100	0.0024	0.67
		250	0.0024	0.23
		500	$6x10^{-4}$	0.99
High mutation number *cis*	Absence of CI	50	0.0028	0.84
		100	0.0045	0.56
		250	0.001	0.99
		500	$6x10^{-4}$	0.99
	Presence of CI	50	0.0031	0.75
		100	0.0027	0.52
		250	0.001	0.99
		500	$9x10^{-4}$	0.96
Low mutation number *trans*	Presence of CI	50	0.0049	0.21
		100	0.0027	0.52
		250	0.0027	0.12
		500	$7x10^{-4}$	0.99
Intermediate mutation number *trans*	Presence of CI	50	0.0034	0.66
		100	0.0017	0.96
		250	0.0015	0.75
		500	$9x10^{-4}$	0.97
High mutation number *trans*	Presence of CI	50	0.0026	0.91
		100	0.0025	0.65
		250	0.002	0.41
		500	$6x10^{-4}$	0.99
