# Supplementary material for: Regulatory network structure determines patterns of intermolecular epistasis
Source: eLife. 2017 Nov 13;6:e28921. doi: 10.7554/eLife.28921 (PMC5699867; doi:10.7554/eLife.28921)
Supplement: Transparent reporting form [file elife-28921-transrepform.pdf]

eLIFE
1st Floor
24 Hills Road
Cambridge CB2 1JP, UK
P 01223 855340
W elifesciences.org
T @elife

eLife's transparent reporting form

We encourage authors to provide detailed information *within their submission* to facilitate the interpretation and replication of experiments. If you have any questions, please contact us: editorial@elifesciences.org.

Sample-size estimation

- You should state whether an appropriate sample size was computed when the study was being designed
- You should state the statistical method of sample size computation and any required assumptions
- If no explicit power analysis was used, you should describe how you decided what sample (replicate) size (number) to use

Please outline where this information can be found within the submission (e.g., page numbers or figure legends), or explain why this information doesn't apply to your submission:

Section 'Single-cell fluorescence measurements' explains in detail how we selected the sample size in our study, and how we verified that this sample size was sufficient to capture phenotypic diversity we report.

Replicates

- You should report how often each experiment was performed
- You should include a definition of biological versus technical replication
- The data obtained should be provided and sufficient information should be provided to indicate the number of independent biological and/or technical replicates
- If you encountered any outliers, you should describe how these were handled
- Criteria for exclusion/inclusion of data should be clearly stated
- High-throughput sequence data should be uploaded before submission, with a private link for reviewers provided (these are available from both GEO and ArrayExpress)

Please outline where this information can be found within the submission (e.g., page numbers or figure legends), or explain why this information doesn't apply to your submission:

The information on replication can be found in the 'Single-cell fluorescence measurements', 'Distributions of single mutation effects in cis- and trans-elements', and 'Combining cis- and trans- element libraries to understand intermolecular epistasis'. This information can also be found in legends to relevant figures.

eLife Sciences Publications, Ltd is a limited liability non-profit non-stock corporation incorporated in the State of Delaware, USA, with company number 5030732, and is registered in the UK with company number FC030576 and branch number BR015634 at the address 1st Floor, 24 Hills Road, Cambridge CB2 1JP | August 2014

Statistical reporting

- Statistical analysis methods should be described and justified
- Raw data should be presented in figures whenever informative to do so (typically when N per group is less than 10)
- For each experiment, you should identify the statistical tests used, exact values of N, definitions of center, methods of multiple test correction, and dispersion and precision measures (e.g., mean, median, SD, SEM, confidence intervals; and, for the major substantive results, a measure of effect size (e.g., Pearson's r, Cohen's d)
- Report exact p-values wherever possible alongside the summary statistics and 95% confidence intervals. These should be reported for all key questions and not only when the p-value is less than 0.05.

Please outline where this information can be found within the submission (e.g., page numbers or figure legends), or explain why this information doesn't apply to your submission:

Statistical tests carried out are described in 'Single-cell fluorescence measurements', 'Distributions of single mutation effects in cis- and trans-elements', 'Predicting system DME', and 'Combining cis- and trans- element libraries to understand intermolecular epistasis'.

(For large datasets, or papers with a very large number of statistical tests, you may upload a single table file with tests, Ns, etc., with reference to page numbers in the manuscript.)

Additional data files ("source data")

- We encourage you to upload relevant additional data files, such as numerical data that are represented as a graph in a figure, or as a summary table
- Where provided, these should be in the most useful format, and they can be uploaded as "Source data" files linked to a main figure or table
- Include model definition files including the full list of parameters used
- Include code used for data analysis (e.g., R, MatLab)
- Avoid stating that data files are "available upon request"

Please indicate the figures or tables for which source data files have been provided:

Source data files are provided for fluorescence measurements done in the plate reader, for the analysis of the effects of single point mutations in cis- and trans-elements (this data is shown in: Figure 2 – Figure Supplement 2). ~1,000,000 single-cell fluorescence measurements used to obtain the distributions of mutational effects are shown in Figure 2, Figure 2 - Figure Supplement 1, Figure 3, and Figure 4. The instrument used for these measurements (FACS Aria II) provides the distributions as the main output, and these outputs were used directly to produce the figures mentioned above.

1st Floor
24 Hills Road
Cambridge CB2 1JP, UK

P 01223 855340
W elifesciences.org
T @elife
